# Predictive Value of Decoy Receptor 3 in Postoperative Nosocomial Bacterial Meningitis

**DOI:** 10.3390/ijms151119962

**Published:** 2014-11-03

**Authors:** Yong-Juan Liu, Li-Hua Shao, Qian Wang, Jian Zhang, Rui-Ping Ma, Hai-Hong Liu, Xiao-Meng Dong, Li-Xian Ma

**Affiliations:** 1 Department of Infectious Diseases, Qilu Hospital of Shandong University, Wenhua Xi Road 107, Jinan 250012, China; E-Mails: gdjuanyl@163.com (Y.-J.L.); GRliuhaihong@126.com (H.-H.L.); GRdongxiaomeng@163.com (X.-M.D.); 2 Department of Laboratory Sciences, School of Public Health of Shandong University, Wenhua Xi Road 44, Jinan 250012, China; E-Mail: Slihuashao@163.com; 3 Department of Clinical Laboratory, Qilu Hospital of Shandong University, Wenhua Xi Road 107, Jinan 250012, China; E-Mails: qianwangql@163.com (Q.W.); zjianql@163.com (J.Z.); 4 Department of Digestive System Diseases, Shandong Provincial Qianfoshan Hospital of Shandong University, Jingshi Road 16766, Jinan 250014, China; E-Mail: maruipingQFS@126.com

**Keywords:** decoy receptor 3, DcR3, bacterial meningitis, diagnosis

## Abstract

Nosocomial bacterial meningitis requires timely treatment, but what is difficult is the prompt and accurate diagnosis of this disease. The aim of this study was to assess the potential role of decoy receptor 3 (DcR3) levels in the differentiation of bacterial meningitis from non-bacterial meningitis. A total of 123 patients were recruited in this study, among them 80 patients being with bacterial meningitis and 43 patients with non-bacterial meningitis. Bacterial meningitis was confirmed by bacterial culture of cerebrospinal fluid (CSF) culture and enzyme-linked immunosorbent assay (ELISA) was used to detect the level of DcR3 in CSF. CSF levels of DcR3 were statistically significant between patients with bacterial meningitis and those with non-bacterial meningitis (*p* < 0.001). A total of 48.75% of patients with bacterial meningitis received antibiotic >24 h before CSF sampling, which was much higher than that of non-bacterial meningitis. CSF leucocyte count yielded the highest diagnostic value, with an area under the receiver operating characteristic curve (ROC) of 0.928, followed by DcR3. At a critical value of 0.201 ng/mL for DcR3, the sensitivity and specificity were 78.75% and 81.40% respectively. DcR3 in CSF may be a valuable predictor for differentiating patients with bacterial meningitis from those with non-bacterial meningitis. Further studies are needed for the validation of this study.

## 1. Introduction

Nosocomial bacterial meningitis is a life-threatening disease, which is more frequently seen in neurosurgical patients (*i.e.*, patients with placement of internal or external ventricular catheters, complicated head trauma, craniotomy) than other patients (patients with spinal anesthesia, myelography, hospital-acquired bacteremia, and so forth) [1]. The incidence rate of nosocomial meningitis can range from 0.8% to 17% following neurosurgical procedure, while its mortality is up to 34% or even higher [1,2,3]. Accurate diagnosis and appropriate treatment are associated with better outcomes [4,5,6].

However, early diagnosis of bacterial meningitis remains a challenge. Clinical and laboratory data are not specific enough. Accurate diagnosis of bacterial meningitis is based on microbiological culture which usually takes at least 24–48 h to yield results and may lead to delayed treatment. Delay in treatment is related to adverse clinical outcome. The microbiological culture results may be negative because of the previous use of antibiotics [1]. For this reason, it is important to identify desirable indicators for the rapid diagnosis of bacterial meningitis.

Decoy receptor 3 (DcR3), a soluble receptor lacking a transmembrane domain, belongs to the tumor necrosis factor (TNF) receptor family and is almost undetectable in serum of normal individuals by enzyme-linked immunosorbent assay (ELISA) [7,8,9]. Levels of DcR3 are elevated under the following diseases or conditions: sepsis [8,9], acute respiratory distress syndrome (ARDS) [10], rheumatoid arthritis [11], and bacterial infection [12]. DcR3 can be a new potential biomarker for inflammatory disease, autoimmune diseases and cancer [13]. One research demonstrates its abundance in human cerebrospinal fluid (CSF) [14]. However, the diagnostic value of DcR3 in bacterial meningitis is not well evaluated.

The aim of present study was to evaluate whether CSF DcR3 was useful in diagnosing nosocomial bacterial meningitis.

## 2. Results and Discussion

### 2.1. Clinical and Biological Characteristics of Patients

The characteristics of the patients with bacterial meningitis and those with non-bacterial meningitis in this study were shown in Table 1. One hundred and twenty-three patients were enrolled for the study, among them 80 patients being diagnosed with bacterial meningitis and 43 patients with non-bacterial meningitis. The mean age (mean ± SD) was 43.15 ± 15.58 years. Of these patients, the rate of receiving antibiotic >24 h before CSF sampling in bacterial meningitis was much higher than that of non-bacterial meningitis (*p* < 0.001). The former rate was as high as 48.75%.

**Table 1:** Clinical features of the patients.

Characteristics	Non-Bacterial Meningitis (*n* = 43)	Bacterial Meningitis (*n* = 80)	*p* Value
Age (years)	42.02 ± 13.90	43.75 ± 16.46	0.560 ^a^
Gender, *n* (%)			0.141 ^b^
Male	25 (58.12)	57 (71.25)	
Female	18 (41.88)	23 (28.75)	
Receiving Steroid >24 h before CSF Sampling	12 (27.91)	27 (33.75)	0.507 ^b^
Receiving Antibiotic >24 h before CSF Sampling	5 (11.63)	39 (48.75)	<0.001 ^b^
Hypertension *n* (%)	7 (16.27)	16 (20.00)	0.614 ^b^
Diabetes *n* (%)	3 (6.98)	6 (7.50)	1.000 ^c^
Leucocyte Count (×10^6^/L)	4.00 (2.00–36.00)	765.00 (265.25–1976.00)	<0.001 ^d^
Glucose (mmol/L)	2.74 (1.59–3.20)	1.59 (0.72–2.58)	<0.001 ^d^
Protein (g/L)	0.45 (0.17–0.79)	0.82 (0.37–1.80)	0.003 ^d^
Lactate (mmol/L)	1.73 (1.11–2.82)	3.23 (1.87–6.42)	<0.001 ^d^
DcR3 (ng/mL)	0 (0–0.192)	0.646 (0.229–1.514)	<0.001 ^d^

All categorical data are expressed as n (%). Quantitative data of normal distribution and non-normal distribution are presented as mean ± SD and median (25th and 75th percentiles) respectively. ^a^: Independent-Samples *t*-Test; ^b^:Pearson Chi-Square Test; ^c^: Continuity Correction; ^d^: Mann-Whitney *U*-Test.

### 2.2. Levels of Decoy Receptor 3 (DcR3) in Patients according to Various Criteria

**Figure 1:**
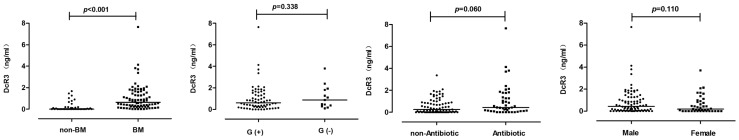
Decoy receptor 3 (DcR3) concentration according to patients’ categories. G (+): Gram-positive bacteria; G (−): Gram negative bacteria; BM: bacterial meningitis; Antibiotic: receiving antibiotic >24 h before CSF sampling.

Concentrations of DcR3 in the patients with bacterial meningitis were significantly higher than those with non-bacterial meningitis (0.646 (0.229–1.514) *vs.* 0 (0–0.192) ng/mL, *p* < 0.001, Table 1 and Figure 1). Among the 80 patients with bacterial meningitis, 79 cases were with a bacterium infection, and one case with mixed bacterial infection (*Staphylococcus haemolyticus* and *Acinetobacter baumanni*) which was classified into the group of Gram-negative bacteria because only Gram-negative bacilli were isolated during subsequent cultures. Levels of DcR3 didn’t differentiate patients with Gram-positive bacteria infection from those with Gram negative bacteria infection (*p* = 0.338) and no significant difference was found between the groups of male and female (*p* = 0.110, Figure 1). DcR3 values were also not associated with treatment of antibiotic/steroid >24 h, hypertension, and diabetes.

### 2.3. Accuracy of DcR3 and Different Cerebrospinal Fluid (CSF) Markers in Diagnosing Bacterial Meningitis

**Figure 2:**
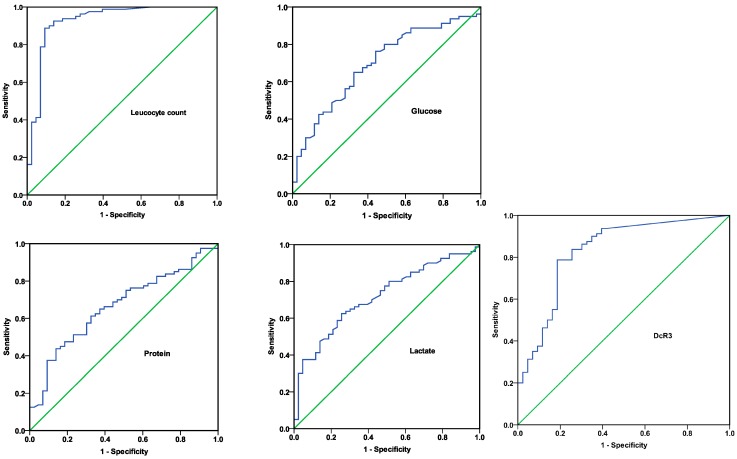
Receiver operating characteristic (ROC) curves of DcR3 and cerebrospinal fluid (CSF) culture markers in predicting bacterial meningitis. All parameters are presented with 95% confidence intervals. Areas under the ROC curve are shown as follows: Leucocyte count: 0.928 (0.872–0.984); Glucose: 0.696 (0.601–0.792); Protein: 0.664 (0.567–0.761); Lactate: 0.717 (0.625–0.808); DcR3: 0.831 (0.752–0.911).

In order to evaluate the diagnostic accuracy of DcR3 and different CSF markers, receiver operating characteristic (ROC) curves and areas under the ROC curve (AUC) were performed. Figure 2 showed that CSF leucocyte count had the best discriminative value, with an AUC of 0.928 (95% Confidence Interval (CI) 0.872–0.984; *p* < 0.001). The AUC of DcR3 in predicting bacterial meningitis was higher than that of CSF biochemistry indicators and lactate. Shown in Table 2 were the sensitivity, specificity, likelihood ratios and predictive values of CSF parameters and DcR3 for the diagnosis of bacterial meningitis. In terms of specificity and sensitivity, CSF leucocyte count was proved to be the best marker.

Multivariate stepwise logistic regression was used to assess possible risk factors with bacterial meningitis. Only CSF DcR3 and protein were found to be the independent predictors for bacterial meningitis (odds ratio (OR) = 5.97, 95% CI = 2.13–16.73, *p* = 0.001 for DcR3; OR = 1.85, 95% CI = 1.05–3.28, *p* = 0.034 for protein; Table 3).

**Table 2:** Diagnostic values of DcR3 and CSF markers for the diagnosis of bacterial meningitis.

Variable	Cut-off Value	Sensitivity (%)	Specificity (%)	PLR	NLR	PPV (%)	NPV (%)
Leucocyte count	116 (×10^6^/L)	88.75 (79.24–94.41)	90.70 (76.95–96.98)	9.54 (3.74–24.34)	0.12 (0.07–0.23)	94.67 (86.19–98.28)	81.25 (66.90–90.56)
Glucose	2.14 (mmol/L)	65.00 (53.44–75.10)	67.44 (51.34–80.46)	2.00 (1.26–3.16)	0.52 (0.38–0.72)	78.79 (66.66–87.52)	50.88 (37.44–64.20)
Protein	1.14 (g/L)	43.75 (32.83–55.27)	86.05 (71.37–94.20)	3.14 (1.43–6.86)	0.65 (0.53–0.80)	85.37 (70.14–93.91)	45.12 (34.24–56.46)
Lactate	2.64 (mmol/L)	62.50 (50.92–72.87)	74.42 (58.53–85.96)	2.44 (1.43–4.18)	0.50 (0.37–0.68)	81.97 (69.60–90.24)	51.61 (38.68–64.34)
DcR3	0.201 (ng/mL)	78.75 (67.89–86.79)	81.40 (66.08–91.08)	4.23 (2.24–7.99)	0.26 (0.17–0.40)	88.73 (78.47–94.66)	67.31 (52.78–79.28)

All parameters except cut-off value are presented with 95% confidence intervals. The Youden Index determines cut-off point. PLR: positive likelihood ratio; NLR: negative likelihood ratio; PPV: positive predictive value; NPV: negative predictive value.

**Table 3:** Multiple logistic regression analysis of factors used for diagnosing bacterial meningitis.

Variable	Coefficient	SE	*p* Value	OR (95% CI)	Chi Square
Glucose	−0.35	0.18	0.047	0.70 (0.50–1.00)	15.83
Protein	0.62	0.29	0.034	1.85 (1.05–3.28)	
DcR3	1.79	0.53	0.001	5.97 (2.13–16.73)	

Pseudo *R*^2^ (Cox and Snell) = 0.275.

### 2.4. Correlation between Age/CSF Parameters and DcR3 Levels

Results of Spearman’s test showed that the correlation between levels of DcR3 and CSF parameters was statistically significant and the concentration of DcR3 was not associated with age (*p* = 0.795). DcR3 was positively correlated with CSF protein and lactate and negatively with CSF glucose (Table 4).

**Table 4:** Correlation between age/CSF parameters and CSF DcR3 concentration.

Variable 1	Variable 2	Spearman’s Rho	*p* Value
DcR3	Leucocyte count	0.696	<0.001
	Glucose	−0.384	<0.001
	Protein	0.296	0.001
	Lactate	0.513	<0.001
	Age	0.024	0.795

### 2.5. Discussion

Results from present study showed that CSF DcR3 might be a useful indicator for the diagnosis of bacterial meningitis. According to the Youden index, the critical value of 0.201 ng/mL for DCR3 to predict bacterial meningitis had a sensitivity of 78.75%, a specificity of 81.40%. ROC analysis indicated that the AUC of DcR3 for discriminating bacterial meningitis from non-bacterial meningitis was 0.831. Although the predictive value of CSF DcR3 levels was high, ROC curve analysis indicated that CSF DcR3 would not be more useful than CSF leucocyte count. CSF Leucocyte count, which yielded a sensitivity of 88.75%, a specificity of 90.70% and an AUC of 0.928, had a better diagnostic value than DcR3 in CSF. However, multiple logistic regression analysis revealed that DcR3 of CSF was an independent risk factor of bacterial meningitis, seemingly better than white blood count. Determination of DcR3 levels in CSF may have additional value to CSF parameters.

CSF levels of DcR3 were elevated in patients with bacterial meningitis compared with those with non-bacterial meningitis. Except for CSF indexes, levels of DcR3 were not associated with gender, age, causative organism, treatment of antibiotic/steroid >24 h, hypertension, and diabetes. Levels of DcR3 were related to low CSF glucose and high CSF protein and lactate levels. A strong positive correlation between levels of DcR3 and counts of white blood cell (WBC) (Spearman’s rho = 0.696; *p* < 0.001) was found in this study. The reasons might be explained as the following: DcR3, like other inflammatory factors (interleukin-8 (IL-8), intercellular adhesion molecule-1 (ICAM-1), *etc.*), has pro-inflammatory function, and can attract leukocytes to phagocytize pathogen after the invasion of bacteria [15,16,17,18,19]; or DcR3 is released from some immune cells [12,20,21]; or both.

Serum levels of some biomarkers such as serum procalcitonin and C-reactive protein (CRP) are important for the diagnosis of bacterial meningitis [22,23]. Research performed by Mueller and partners has found that average DcR3 level of CSF is much higher than that of serum under various neurological disease states [14]. Because DcR3 levels in sera were not determined in present study, diagnostic significance of DcR3 in blood for bacterial meningitis was not evaluated. Further studies are needed to assess the potential role of serum DcR3 for the diagnosis of bacterial meningitis.

Major limitations in present study were as follows: (1) This study was retrospective and the sample size was not satisfactorily large; (2) Patients included in the group of bacterial meningitis were only those with positive results of bacterial culture. Negative CSF cultures occur in 11%–30% of patients with bacterial meningitis [24]. Some patients with non-bacterial meningitis might be misclassified; (3) Detection of DcR3 was just at one point. Hence, dynamic changes of CSF DcR3 levels in predicting severity and prognosis of bacterial meningitis were not observed. Further studies, especially prospective studies of collecting serum and CSF samples at multiple time points, are warranted to substantiate our findings.

## 3. Experimental Section

### 3.1. Patients and Data Collection

All patients in this study were enrolled in Qilu Hospital of Shandong University in China between November 2012 and October 2013. Included in the clinical features were age, gender, hypertension, diabetes, received antibiotic/steroid >24 h before CSF sampling, CSF glucose, protein, lactate and microorganism confirmed by CSF culture. Laboratory analyses of CSF and CSF culture were performed in emergency laboratory and microbiology laboratory respectively.

According to laboratory data and microbiological data, all patients in present study were divided into two groups. Group one of bacterial meningitis consisted of patients with a positive culture of CSF (*n* = 80). Patients with coagulase-negative Staphylococci infection were defined with stricter criteria in which repeated CSF cultures were positive or CSF leucocyte count >100 × 10^6^/L with >50% neutrophils. Group two of non-bacterial meningitis chose patients with a negative CSF culture, plus CSF leucocyte count <50 × 10^6^/L and neutrophils <50% (*n* = 43).

Patients or clients refused to participate in the study were excluded. Ethical approval was obtained on 11 October 2012, from Institutional Research Ethics Committee of Qilu Hospital of Shandong University (No. KYLL-2012 (KS)-096). Written informed consents were taken from patients or clients before registration.

### 3.2. Detection of DcR3

The CSF samples were frozen at −80 °C until assay. CSF levels of DcR3 were determined in duplicate by ELISA according to the kits instructions (Cusabio, Wuhan, China). The lower limit of detection was 0.039 ng/mL. Intra-assay coefficient of variation was <8% and inter-assay coefficient of variation was <10%.

### 3.3. Statistical Analysis

Descriptive results of age were expressed as mean ± SD and the other continuous data were expressed as median (25th and 75th percentiles). Continuous data were compared with independent-samples *t-*test or Mann–Whitney *U*-test. Chi-square test or continuity correction were used to analyze categorical variables. The association between DcR3 levels and clinical or biological features was done by Spearman’s correlation test. ROC curve and AUC were constructed to illustrate the diagnostic accuracy of DcR3 and different CSF markers in differentiating bacterial meningitis from non-bacterial meningitis. Cut-off CSF value was defined by the Youden index (*J* = max (sensitivity + specificity − 1)). Risk factors related to bacterial meningitis were explored by multivariate stepwise logistic regression analysis. Statistical analyses were carried out with SPSS, version 20.0 and a two-sided *p* < 0.05 was considered to be statistically significant.

## 4. Conclusions

In conclusion, DcR3 levels were elevated in bacterial meningitis patients and may act as an indicator of bacterial meningitis. Detection of DcR3 in CSF provides convenience and high accuracy, but additional confirmatory research is needed before clinical application.
